# Photocatalytic Deposition of Au Nanoparticles on Ti_3_C_2_T_x_ MXene Substrates for Surface-Enhanced Raman Scattering

**DOI:** 10.3390/molecules29102383

**Published:** 2024-05-18

**Authors:** Zhi Yang, Lu Yang, Yucun Liu, Lei Chen

**Affiliations:** 1 College of Chemistry, Jilin Normal University, Siping 136000, China; yangzhi12260102@163.com (Z.Y.); yanglu4818@163.com (L.Y.); 2 School of Materials Science and Engineering, Jilin Jianzhu University, Changchun 130118, China

**Keywords:** MXene, Au NPs, photocatalytic, SERS, carbendazim

## Abstract

Surface-enhanced Raman scattering (SERS) is a promising technique for sensitive detection. The design and optimization of plasma-enhanced structures for SERS applications is an interesting challenge. In this study, we found that the SERS activity of MXene (Ti_3_C_2_T_x_) can be improved by adding Au nanoparticles (NPs) in a simple photoreduction process. Fluoride-salt-etched MXene was deposited by drop-casting on a glass slide, and Au NPs were formed by the photocatalytic growth of gold(III) chloride trihydrate solutions under ultraviolet (UV) irradiation. The Au–MXene substrate formed by Au NPs anchored on the Ti_3_C_2_T_x_ sheet produced significant SERS through the synergistic effect of chemical and electromagnetic mechanisms. The structure and size of the Au-decorated MXene depended on the reaction time. When the MXene films were irradiated with a large number of UV photons, the size of the Au NPs increased. Hot spots were formed in the nanoscale gaps between the Au NPs, and the abundant surface functional groups of the MXene effectively adsorbed and interacted with the probe molecules. Simultaneously, as a SERS substrate, the proposed Au–MXene composite exhibited a wider linear range of 10^−4^–10^−9^ mol/L for detecting carbendazim. In addition, the enhancement factor of the optimized SERS substrate Au–MXene was 1.39 × 10^6^, and its relative standard deviation was less than 13%. This study provides a new concept for extending experimental strategies to further improve the performance of SERS.

## 1. Introduction

Surface-enhanced Raman scattering (SERS), a highly sensitive and efficient characterization method, provides rich and ultra-high-resolution structural information about the analyte, down to the single-molecule level. This enables the rapid detection and characterization of targets. Owing to its sensitivity and stability, SERS can be used in the fields of food safety, national defense, environmental monitoring, biology, biomedicine, etc. [1,2,3,4]. To fabricate SERS substrates with excellent performance, a variety of new materials, such as precious metals, semiconductors, and organic compounds, have been fabricated and investigated. Plasmonic structures formed by the assembly of Au or Ag NPs have been extensively investigated for their ability to combine incident and electromagnetic fields at the metal surface with localized surface plasmon resonance to achieve SERS enhancement. Although the contribution of the chemical mechanism (CM) to Raman signal enhancement is considered much smaller than that of the electromagnetic mechanism (EM), it is still of great significance owing to the potential for chemical interactions between the plasma metal surface and adsorbed molecules, which can enable surface plasma-enhanced photochemical reactions [5]. At present, SERS is particularly problematic owing to the generation of interference signals caused by the non-reproducible aggregation of NPs and the change in adsorption molecules and functional groups on the substrate surface during laser excitation. With these challenges, emerging strategies have evolved from easily dispersed noble-metal NPs to the construction of large-area and highly SERS-active NPs through the controllable chemical modification of the surfaces of 2D materials.

As novel and promising 2D nanomaterials, MXenes are based on carbides, carbon nitrides, and nitrides [6]. The rapidly emerging 2D materials have attracted much attention due to their excellent hydrophilicity, high metal conductivity, tunable electronic structure, low-cost manufacturing process, and good flexibility. MXenes with large surface areas and abundant surface defects can be used as matrices for multifunctional metal decoration [7]. Among them, Ti_3_C_2_T_x_ MXene (where T_x_ represents surface functional groups such as –OH, –F, and –O) [8] stands out owing to its better chemical stability, advanced conductivity, and remarkable focusing ability [9,10], and it has therefore been prepared and applied in various fields [11,12,13,14,15,16,17,18,19]. In particular, the sensitive monitoring of SERS has aroused significant attention. For example, Yu et al. reported the preparation of self-assembled MXene nanosheets/Au nanostructures to enhance the sensitivity of SERS application through plasma coupling and charge transfer (CT). Although the dispersion of MXene nanosheets was not uniform, it could still be used to detect R6G, methylene blue (MB), and other dye molecules [20]. Li and his group deposited the Au NPs on defective TiO_2_ nanosheets and detected MB to reveal the enhancement of substrate–molecule vibration coupling and especially the SERS mechanism in light-induced processes [21].

The carbendazim (CBZ) has high insecticidal efficiency and low cost, and it is widely used in various fungal diseases afflicting crops, such as drought, the yellowing of leaves, and white mushroom growth on fruit trees [22]. Farmers tend to use excessive amounts of insecticides to ensure adequate pest and disease control for crop protection. However, the abuse of CBZ leads to high levels of residues in agricultural products and soils. Simultaneously, the presence of CBZ may endanger the lives and health of humans and animals, leading to reproductive and fetal development problems [23,24]. Therefore, developing a highly sensitive method for the determination of pesticide pollutants is imperative. SERS, as an advanced technique, was considered to be a sensitive and selective method for the qualitative and quantitative analysis of CBZ. In recent decades, many research teams have conducted SERS research on different substrates to promote the development of accurate detection of CBZ. Zhai et al. prepared the ITOAu@Ag@Ag@ZIF chip by fixing organic ligands and metal ions or clusters on ITO glass through coordination bonds and used SERS to realize the rapid detection of CBZ with a concentration of 10^−9^ mol/L in seawater, which has high stability and detection sensitivity [25]. Ma et al. selected Au colloid to detect the concentration of CBZ in tea using the SERS method. The detection limit was 0.1 mg/kg, and its recovery rate in tea samples was 72.3%, which was helpful to study the half-life of tea [26]. Luong et al. successfully fabricated the zinc oxide nanorods (ZnO NRs) decorated with polymorphic Ag NPs on polydimethylsiloxane (PDMS) films to form a three-dimensional stretchable structure and used it for CBZ detection at 10^−3^ ppm. The detection limit (LOD) reached high performance at low concentrations [27]. Although the detection and analysis of these SERS substrates are reliable and accurate, the preparation of the substrates is still complicated, time-consuming, costly, and difficult to operate. Therefore, in order to solve the problems existing in the substrate, a simple, rapid, and real-time substrate preparation method should be developed.

In this study, we prepared MXene (Ti_3_C_2_T_x_) by fluoride etching and used a simple and rapid photochemical method to modify Au NPs on the surface of the MXene nanosheets without using a reducing agent. The Au–MXene substrate was prepared through the UV catalytic deposition of Au NPs. The large surface area of the MXene is beneficial for the deposition of Au on MXene glass slides and the adsorption of the detected substances. By tuning the photoreduction time of the Au precursors, the hot spots on the MXene surface could be controlled, which increased the SERS activity of the Au–MXene substrate. The new metal–2D material composite employed for the sensitive monitoring of CBZ achieved a high detection limit and reproducibility, indicating countless possibilities for practical applications.

## 2. Results and Discussion

**Figure 1:**
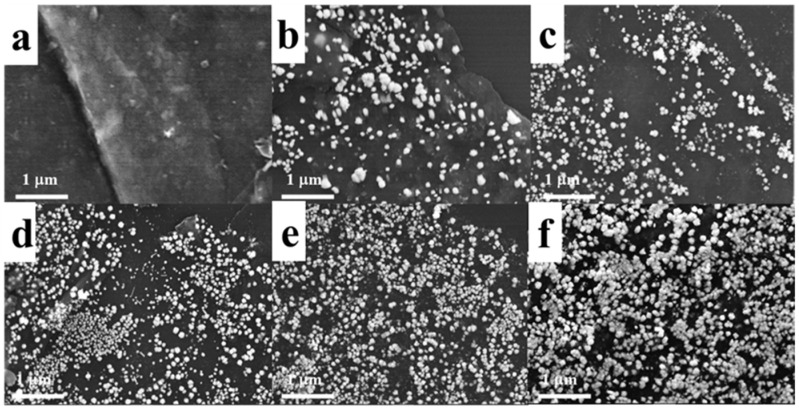
SEM images of Au–MXene composites prepared after photoreduction times of (**a**) 0, (**b**) 10, (**c**) 20, (**d**) 30, (**e**) 40, and (**f**) 50 min.

The morphologies of the MXene (Ti_3_C_2_T_x_) nanosheets and Au–MXene composites were analyzed using SEM. Figure 1a shows the SEM image of the pure MXene (Ti_3_C_2_T_x_) prepared on a glass sheet, revealing a 2D membrane structure with a large surface area. Its surface is relatively smooth and has no obvious particles. It can be seen that MXene prepared using the drip method formed a thin slice shape on the glass plate. Compared with MXene obtained by self-reduction, it is easier for the thin slices to be stacked so that MXene obtained by the drip method looks a bit thicker. Figure 1b–f depict SEM images of the Au–MXene composites prepared after varying photoreduction times (10, 20, 30, 40, and 50 min). It is clear to show that the Au NPs appeared unevenly on the surface of the MXene. With an increase in the photoinduced reduction time, the Au NPs appeared, gradually increased in density, and finally aggregated. As can be seen in the image, a large number of Au NPs were successfully decorated on the surface of the MXene (Ti_3_C_2_T_x_) through photoreduction. With the increase in photoreduction time, the density of the Au NPs on the surface of Ti_3_C_2_T_x_ increased significantly. However, when the photoreaction time is between 40 and 50 min, the size and density of the Au NPs increase significantly. Simultaneously, the particle sizes of the Au NPs at different reaction times were measured, as shown in Figure S1 (Supplementary Materials). 

**Figure 2:**
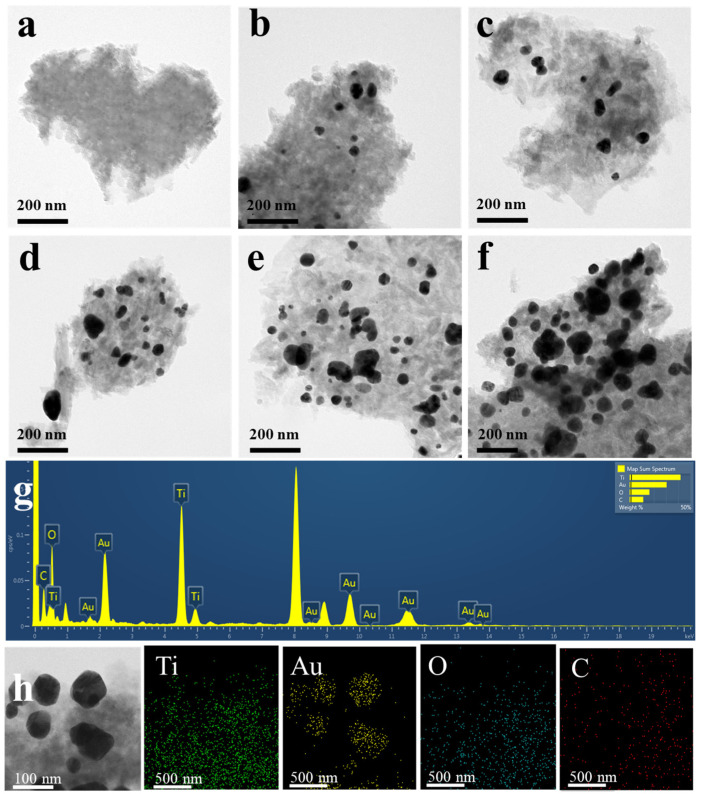
TEM images of Au–MXene composites with varying photoreaction times with the Au precursor on the MXene sheet: (**a**) 0, (**b**) 10, (**c**) 20, (**d**) 30, (**e**) 40, and (**f**) 50 min. (**g**) EDS of the Au–MXene and (**h**) TEM–EDS elemental mapping images corresponding to Ti, Au, O, and C.

TEM was used to more closely observe the morphology of the MXene (Ti_3_C_2_T_x_) nanosheets and Au–MXene composites prepared after varying photoreduction times of 0, 10, 20, 30, 40, and 50 min (Figure 2a–f). The MXene (Ti_3_C_2_T_x_) nanosheets exhibited an ultrathin layered structure (Figure 2a). Under photoreducing conditions (0, 10, 20, 30, 40, and 50 min), the MXene (Ti_3_C_2_T_x_) sheets were successfully decorated with Au NPs. Figure 2 shows that when the photoreaction time was increased from 0 to 50 min, the size, density, and degree of aggregation of Au NPs were gradual (Figure 2b–f). With an increase in the photoreaction time, the size of most Au NPs increased, and the Au NPs agglomerated significantly. Elemental mapping images were obtained using TEM and EDS (Figure 2g,h). C, O, Ti, and Au were evident in the elemental mapping images of the composite. The preparation of the MXene (Ti_3_C_2_T_x_) was verified by the elemental distribution and energy peaks corresponding to Ti, C, and O. The energy peaks corresponding to Au were located at 2.20, 9.71, and 11.47 keV and were mainly attributed to Au-Mβ, Au-Lα, and Au-Lβ [28], respectively, confirming that the MXene (Ti_3_C_2_T_x_) was successfully decorated with the Au NPs.

**Figure 3:**
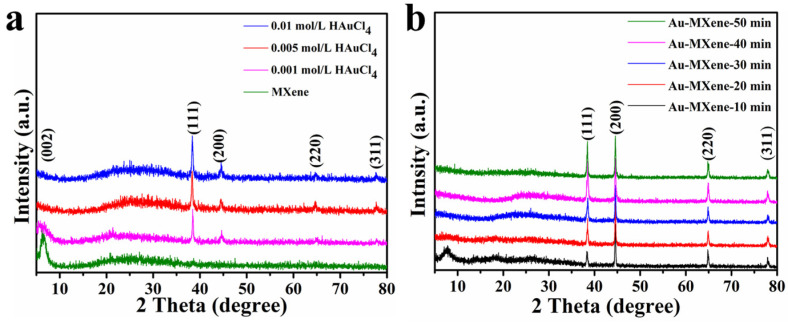
XRD patterns of the MXene nanosheets and Au–MXene composites for (**a**) varying concentrations of the Au precursor (0.01, 0.005, and 0.001 mol/L) and (**b**) varying photoreaction times (10, 20, 30, 40, and 50 min).

XRD was employed to further confirm the successful preparation and crystal structure of the MXene nanosheets and Au–MXene composites, as shown in Figure 3. We observed a peak around 8.9°, indicating the formation of the lamellar structure of the MXene. After the decoration of the Au NPs, the four main diffraction peaks of the (111), (200), (220), and (311) planes reacted to the face-centered cubic Au single crystal. For the same photoreduction time, the characteristic peak intensity of the Au NPs increased with increasing HAuCl_4_ concentration, and when the concentration of the Au precursor was 0.01 mol/L, the particles agglomerated due to the high concentration of Au NPs, and there were no more obvious changes to the characteristic peak (Figure 3a). In Figure 3b, the Au peaks clearly sharpen and intensify with an increasing reaction time, up to 40 min, indicating enhanced crystallinity of the Au NPs. Afterward, the peak intensity underwent no obvious change, and the peak width increased slightly, which was due to the effect of particle agglomeration. In addition, the intensity of the (002) peak of the MXene nanosheets at ≈8.9° weakened, which was presumed to be caused by the partial oxidation of the MXene (Ti_3_C_2_T_x_) nanosheet flakes during the reaction of the Au NPs [29].

**Figure 4:**
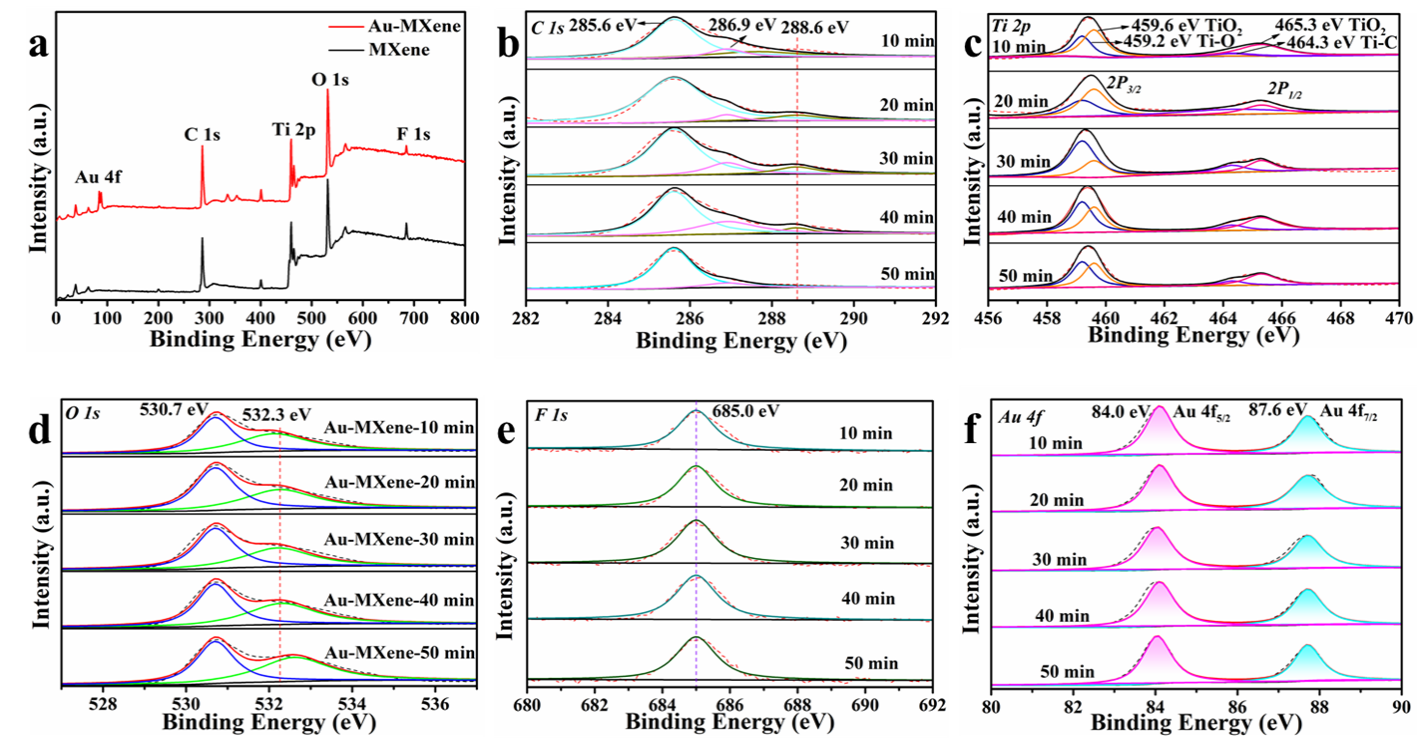
(**a**) Survey XPS spectra of MXene nanosheets and Au–MXene composites, and XPS spectra of (**b**) C, (**c**) Ti, (**d**) O, (**e**) F, and (**f**) Au in Au–MXene composites at varying photoreaction times (10, 20, 30, 40, and 50 min).

In order to clarify the chemical composition of the prepared materials, XPS was used to analyze the MXene (Ti_3_C_2_T_x_) nanosheets and different Au–MXene composites. The survey XPS spectra and XPS spectrum of the Ti 2p, C 1s, O 1s, and F 1s of the MXene (Ti_3_C_2_T_x_) nanosheets are shown in Figure 4. According to the survey XPS spectra in Figure 4a, the –O and –F groups were introduced during the reaction process, while Al was etched away. XPS spectra of all the elements associated with the Au–MXene composites (C 1s, Ti 2p, O 1s, F 1s, and Au 4f) were observed at varying times. The C 1s peaks of the Au–MXene composites were fitted to the three component peaks at 285.6, 286.9, and 288.6 eV (Figure 4b), corresponding to C–O, C=O, and O–C=O, respectively, and with an increasing photoreaction time, the peak at 288.6 eV disappeared owing to its own partial reduction reaction [30]. Similarly, in the Ti 2p spectra, the Ti–O, TiO_2_, and Ti–C bond energies were 459.2, 459.6 (465.3 eV), and 464.3 eV, respectively (Figure 4c) [31]. The different samples exhibited similar bond energy positions, indicating that the basic skeletal framework of the material did not change. The O 1s spectra (Figure 4d) were deconvoluted into two peaks at 530.7 and 532.3 eV, which represent lattice oxygen and surface-adsorbed oxygen, respectively. The surface oxygen adsorption peak (523.3 eV) shifted as the UV irradiation time increased. This is due to the conversion of the carbonyl group on the surface of MXene to the hydroxyl group, resulting in an increase in the number of hydroxyl functional groups in the composite and further resulting in an oxidation reaction. The F 1s peak (Figure 4e) at 685.0 eV was attributed to C–Ti–F and did not appear to change with reaction time, indicating that F did not participate in the reaction and that this surface functional group was stable. The appearance of Au 4f (Figure 4f) indicates that Au was successfully modified on the MXene (Ti_3_C_2_T_x_) nanosheets. The characteristic peaks of the Au–MXene composites (Figure 4f) at 84.0 eV (Au 4f_7/2_) and 87.6 eV (Au 4f_5/2_) confirm the presence of Au NPs in the Au–MXene composites. The binding energies, which differ by 3.6 eV, indicate the reduced form of Au (i.e., Au(0)), proving that all samples contained Au. 

**Figure 5:**
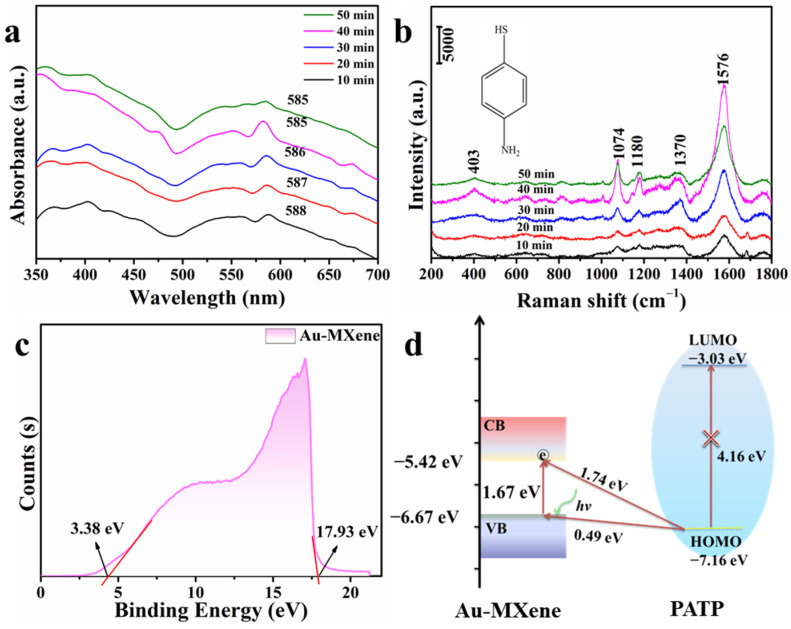
(**a**) UV–Vis absorbance spectra of the MXene nanosheet and Au–MXene substrates formed after varying photoreaction times (10, 20, 30, 40, and 50 min). (**b**) SERS spectra of PATP adsorbed on the Au–MXene after varying photoreaction times (10, 20, 30, 40, and 50 min) under 633 nm laser excitation. (**c**) UPS spectra of Au–MXene and (**d**) a schematic of the energy levels of the Au–MXene–PATP composites.

The UV–Vis absorbance spectra were observed based on the transmittance method. The UV–Vis absorbance spectra of the MXene (Ti_3_C_2_T_x_) nanosheets and Au–MXene composites at varying photoreaction times (10, 20, 30, 40, and 50 min) are shown in Figure 5a and Figure S2. The absorbance peak at ≈310 nm is mainly attributed to the self-coupling vibration of the MXene (Ti_3_C_2_T_x_) nanosheets (Figure S2). However, this peak was red-shifted to 340 nm after the addition of Au NPs, which was caused by the redistribution of the electrons in the Au–MXene composites. With the decoration of Au NPs on the MXene nanosheets, a characteristic peak at around 580 nm could be assigned to the SPR peak of Au NPs, and the slightly increased absorbance peak indicates the increase in the density of Au NPs. The band gap of the Au–MXene composites was analyzed using the Kubelka–Munk formula combined with UV–Vis absorbance spectroscopy, as follows [32]:

(1) $${\left(\frac{Ahv}{k}\right)}^{\frac{1}{2}}=hv-E_{g}$$

where *hv* is the photon energy, *A* is the absorbance, and *k* is an *E_g_*-independent constant. The plot of (*Ahv*)^1/2^ vs. the photon energy (*hv*) in Figure S3 shows that the Au–MXene composites are tangential to the ste88epest point. The band gap of the Au–MXene composites was calculated to be 1.25 eV. The band gap of the Au–MXene composites was smaller than that of MXene nanosheets, which confirms that the photocatalytic method enables Au nanoparticles to be generated completely on the surface of MXene nanosheets.

Owing to the special surface defects and abundant surface functional groups of MXene, electrons can participate in CT and adsorption between nanosheets and metal and probe molecules. Figure S4 shows SERS spectra of adsorbed 4-aminophenthiophenol (PATP, 10^−3^ mol/L) on the MXene and Au–Mxene 30 min composite. It is obvious that the SERS intensity of PATP on the Au–MXene composites was higher than that of MXene, especially the SERS peak at 1576 cm^−1^. Due to the large specific surface area of MXene, a large number of Au NPs were adsorbed on the surface of MXene. Thus, higher SERS intensity was observed due to the contribution of the SPR of Au NPs. On the other hand, the rich carriers of Au NPs enhance CT, and the addition of Au NPs provides another possibility for CT in the system. To determine the contribution of CT, we calculated its degree, which was 0.57 (Figure S5), confirming the synergistic effect of SPR and CT.

To analyze the SERS activity of the proposed Au–MXene composite substrates prepared after varying photoreaction times, PATP (10^−3^ mol/L) was used as the probe molecule. Figure 5b displays the SERS spectra of PATP adsorbed on the Au–MXene composites. The assignments of the characteristic SERS bands of PATP are summarized in Table S1 [33]. In the spectra, the a_1_ modes at 1074 and 1180 cm^−1^ were assigned to C–S stretching and C–H bending, and the b_2_ mode at 1576 cm^−1^ was linked to C=C stretching. Comparing the characteristic SERS band intensities of PATP adsorbed on the Au–MXene composites at varying times (10, 20, 30, 40, and 50 min), the SERS intensity initially increased and then decreased. The highest SERS intensity occurred for the PATP adsorbed onto the Au–MXene composites at a reaction time of 40 min and could be attributed to both the EM and CM. The EM involves the enhancement of the localized electromagnetic field provided by the hotspot of the Au NPs aggregate, whereas the CM originates from the CT and chemical bonding effects between the MXene (Ti_3_C_2_T_x_) nanosheets, the Au NPs, and the probe molecules. In the EM for the Au NPs, the number of Au NPs increased accordingly, resulting in a strong electromagnetic field. With the aggregation of Au NPs, an increase in the aggregates leads to a significant weakening of the surface electromagnetic field [34]. To further illustrate the contribution of EM, we tested the carriers. Hot electron conduction was difficult owing to particle agglomeration; therefore, the carrier concentration first increased and then decreased (Figure S5a). Undoubtedly, the carrier concentration affects the SPR of the system, which weakens the EM contribution of the system when the reaction time reaches 50 min. For the CM, the enhancement of the SERS signal was dependent upon the number of analytes present in the measured area [35]. Due to the difference in dielectric defects, before the new electric field “hot spot” is generated at the interface between the MXene (Ti_3_C_2_T_x_) nanosheet and the Au NPs, the SPR effect of the light-excited Au NPs is dominant, inducing the CT. 

To explore the enhancement effect of the Au–MXene substrate, the enhancement factor (EF) was calculated in detail according to a previous method [36]. The intensity of the peak at 1074 cm^−1^ of PATP was chosen for the calculation, and the EF was calculated to be 1.39 × 10^6^.

The enhancement mechanism was investigated using the bands at 1074 and 1576 cm^−1^ assigned to C–S stretching (a_1_ mode), as well as the peaks at 1370 and 1180 cm^−1^ assigned to incomplete C–C stretching (b_2_ mode). Notably, the energy band intensities of the peaks increased by a similar multiplicity for different SERS substrates. In other words, the vibration peak of PATP was selectively and synchronously enhanced, indicating a new *CT* process through the Herzberg–Taylor contribution [37]. The charge-transfer degree (*ρ_CT_*) was employed to evaluate the *CT* contribution [38,39]:

(2) $$\rho_{CT}=\frac{\frac{b_{2}}{a_{1}}}{1+\frac{b_{2}}{a_{1}}}$$

where *b*_2_ is a non-perfectly symmetric vibration at 1180 cm^−1^, and *a*_1_ is a perfectly symmetric vibration at 1074 cm^−1^. Thus, the *ρ_CT_* values calculated for the Au–MXene systems were 0.47, 0.50, 0.57, 0.62, and 0.61 (Figure S5b). This shows that the *ρ_CT_* decreased slightly with a reaction time of 50 min, which was due to the difficulty of CT because of particle aggregation.

UV photoelectron spectroscopy (UPS) was used to evaluate the conduction band (CB) and valence band (VB) of the Au–MXene substrates (Figure 5c). The band gap of the Au–MXene composite can be calculated, and the CB and VB were calculated to be −5.42 eV and −6.67 eV, respectively. As shown in Figure 5d, the CT processes of the Au–MXene composite and PATP system were analyzed. According to the UV–Vis absorbance spectra, the *E_g_* value was calculated to be 1.25 eV. An excitation wavelength of 633 nm (1.96 eV) was used for SERS [40]. According to a previous report, the highest occupied molecular orbital (HOMO) and lowest occupied molecular orbital (LUMO) of PATP were calculated to be −7.16 eV and −3.03 eV, respectively [41]. The electron in the HOMO of PATP was excited to the CB of the Au–MXene composite by SPR induction, and then the electrons in the VB were excited to the CB of the Au–MXene composite based on 633 nm laser excitation. 

**Figure 6:**
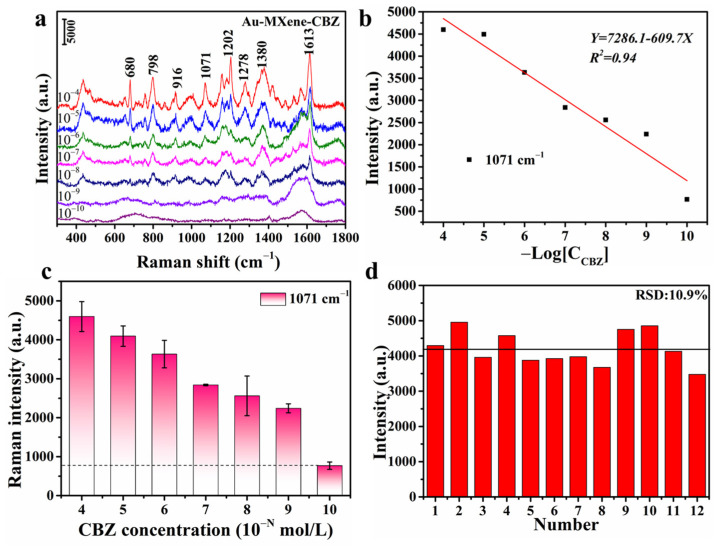
(**a**) Concentration-dependent SERS spectra of Au–MXene composites (40 min), and (**b**,**c**) relationship between the CBZ concentration and SERS intensity in 1071 cm^−1^. The error bars indicate the standard deviations from three independent measurements. (**d**) Histogram of the intensity distribution at the 1071 cm^−1^ peak in the 12 spectra of the Au–MXene substrate (1 × 10^−5^ mol/L).

As shown in Figure 5b, the Au–MXene produced at a photoreaction time of 40 min exhibited the best SERS activity. Thus, we employed the proposed Au–MXene substrate for the quantitative determination of CBZ. CBZ is a benzimidazole fungicide frequently used for leaf spraying and seed and soil treatment. The toxicity of CBZ to mammals is low, but in high quantities, it can damage their reproduction and growth. We employed the Au–MXene substrate as the SERS platform for the quantitative analysis of the CBZ in the concentration range of 10^−4^–10^−10^ mol/L, as shown in Figure 6a. As the CBZ concentration decreased, the SERS intensity also progressively decreased. The characteristic peaks at 798 and 1071 cm^−1^ can be attributed to the C–O–CH_3_ and C–N bending vibrations, respectively, and the peak at 1380 cm^−1^ is attributed to the C–N stretching vibration. The typical vibrational frequencies and modes of the CBZ molecule are listed in Table S2 [42]. The main characteristic peak of CBZ at 1071 cm^−1^ was selected to monitor the change in SERS intensity with CBZ concentration. However, the characteristic SERS peak of CBZ was observed when the concentration was reduced to 10^−9^ mol/L. Interestingly, the SERS intensity and CBZ concentration exhibited a linear relationship, indicating that the quantitative evaluation of this analyte was feasible (Figure 6b). The calibration plot showed a good linear response, with a linear regression equation of I_1071_ = 609.7 log [CBZ] + 7286.1. In addition, a good linear relationship existed between the peak intensity and the negative logarithm of the concentration, with a linear correlation coefficient (R^2^) of 0.94. The limit of detection (LOD) of CBZ adsorbed on the Au–MXene composite was evaluated (Figure 6c) [43,44]. With decreasing CBZ concentration, the SERS intensity decreased continuously. The error bars represent the standard deviations among the three groups of parallel-measured values. When the CBZ concentration was 10^−9^ mol/L, the SERS signal was easy to identify. When the concentration was 10^−10^ mol/L, the SERS intensity was below the threshold line; thus, the signal could not be effectively detected. The detection limit was 10^−9^ mol/L, confirming that the proposed Au–MXene substrate exhibited excellent sensitivity. The Au–MXene composite showed excellent sensitivity, an extremely low detection limit, and stable reproducibility for CBZ detection, which can greatly facilitate the practical monitoring of this composite material when applied to CBZ pesticides.

To evaluate the reproducibility of the Au–MXene composites, we randomly selected 12 spots on 10^−5^ mol/L of CBZ-adsorbed Au–MXene composites. All the spectra showed characteristic peaks consistent with Figure S6. The SERS intensities of CBZ were consistent. To investigate the reproducibility of SERS activity, the relative standard deviation (RSD) was calculated by applying the corresponding characteristic peaks of CBZ, as shown in Figure 6d. The strongest characteristic peak of CBZ appeared at 1071 cm^−1^, and the RSD of CBZ was 10.9%, indicating that the Au–MXene SERS substrate had excellent reproducibility.

## 3. Materials and Methods

### 3.1. Preparation of MXene (Ti_3_C_2_T_x_) Nanosheets and Au–MXene Substrate

Titanium–aluminum carbide powder (Ti_3_AlC_2_) at 200 mesh/100 g was purchased from 11 Science and Technology Co (Changchun, China). Hydrochloric acid (HCl, 35–38%) was purchased from Chengdu Cologne Chemical Co (Chengdu, China). Lithium fluoride (LiF), hydrochloroauric acid (HAuCl_4_), and p-mercaptoaniline (PATP) were purchased from Aladdin (Shanghai, China). Ultrapure water (≥18.2 MΩ·cm, 25 °C) was used for all experiments.

Lithium fluoride (2 g) was mixed with concentrated hydrochloric acid (36%) and 10 mL of deionized water, and the solution was stirred for 5 min at 25 °C to fully dissolve the salt. A total of 1 g of the MXene (Ti_3_C_2_T_x_) precursor (Ti_3_AlC_2_) was added to this solution and kept in a water bath. The mixture was washed and centrifuged. This was repeated several times until the pH reached 7. The precipitate was sonicated in water, centrifuged for 30 min, and then freeze-dried. Single or multiple layers of MXene (Ti_3_C_2_T_x_) were obtained as a powder.

**Scheme 1:**
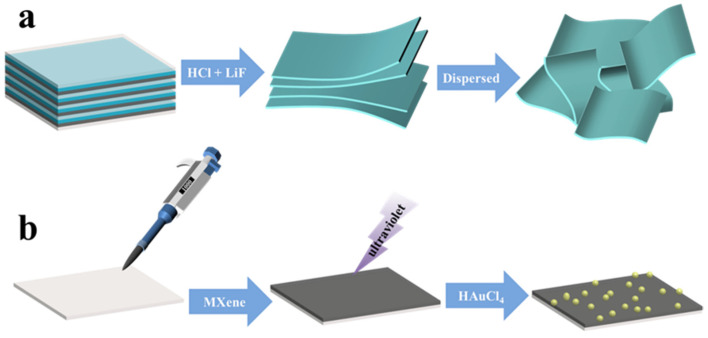
Schematic of the preparation process of (**a**) MXene (Ti_3_C_2_T_x_) nanosheets and (**b**) Au–MXene substrates.

The Au–MXene substrates were prepared in two steps (Scheme 1). Firstly, 150 µL of the 1 g/L MXene (Ti_3_C_2_T_x_) suspension was dripped onto a glass sheet and dried. Next, the MXene-coated glass slide was immersed in 0.005 mol/L of HAuCl_4_ solution and irradiated with UV light. After the reaction was completed, the slide was washed several times with deionized water to remove the independently Au particles, and then it was dried at room temperature to obtain the prepared Au–Ti_3_C_2_T_x_ MXene composites. 

### 3.2. SERS Measurement

PATP molecules were chosen as probes to analyze the SERS properties of the Au–MXene substrate. The Au–MXene substrate was immersed in 200 μL of PATP solution (10^−3^ mol/L) for 30 min, then the PATP-decorated Au–MXene substrate was washed with anhydrous ethanol.

### 3.3. Characterization

The morphology and microstructure of the Au–MXene substrates were observed using a JEOL 6500F scanning electron microscope (SEM) manufactured by Nippon Electron Co (Tokyo, Japan). Ultraviolet–visible (UV–Vis) absorbance spectra were obtained using a Shimadzu UV-3600 spectrometer (Kyoto, Japan). Transmission electron microscopy (TEM) and energy-dispersive X-ray spectra (EDS) were acquired using a JEOL instrument by Nippon Electron Co (Tokyo, Japan). X-ray diffraction (XRD) analysis was performed at 25 ℃ using a Rigaku D/max-ga X-ray diffractometer equipped with a Cu Kα radiation source (λ = 1.5418 Å) by using the Bruker Smart Apex II (Saarbrucken, Germany). The step size (2θ) was 0.01. X-ray photoelectron spectroscopy (XPS) was performed using an Excalab 250Xi X-ray spectrometer (Thermo Fisher Scientific, Waltham, MA, USA). UV photoelectron spectroscopy (UPS) was performed using a Polish PREVAC XPS/UPS system (Warsaw, Poland). All Raman spectra were obtained using a Confocal Raman System 2000 microscope spectrometer equipped with a 532 nm laser (Renishaw Co., London, UK). The optical power of the excitation condition was 20 mW, and the acquisition time was 30 s.

## 4. Conclusions

In summary, Au–MXene substrates were prepared using UV photoreduction to modulate the density and size of the Au NPs by varying the content of HAuCl_4_ with different photoinduced reaction times. As a result of the synergistic effect of the Au NPs and MXene nanosheets, the EM and CM produced a strong SERS effect, leading to excellent SERS activity and good reproducibility. Therefore, the Au–MXene composites can be used as SERS substrates for the ultrasensitive detection of CBZ. The Au–MXene composites showed good sensitivity for the analysis of CBZ, with good reproducibility and liner correlation; the LOD was as low as 10^−9^ mol/L. Importantly, this research opens a new avenue for the preparation and application of metal–MXene composites in various fields and provides a new method for the detection of organic pesticides.

## Data Availability

Data are available in a publicly accessible repository.

## Supplementary Materials

The following supporting information can be downloaded at: https://www.mdpi.com/article/10.3390/molecules29102383/s1, Figure S1: The size distribution of Au NPs absorbed on the surface of MXene nanosheets at different photoreaction times (10, 20, 30, 40, and 50 min); Figure S2: UV–Vis absorption spectra of MXene and Au–MXene composites (10, 20, 30, 40, and 50 min); Figure S3: The plot of (Ahv)^1/2^ versus photon energy (hv); Figure S4: SERS spectra of PATP adsorbed on the MXene and Au–MXene composites. Figure S5: The photoreaction time of Au–MXene composites depends on the relationship between (a) carrier concentration and (b) charge transfer degree; Figure S6: SERS spectra CBZ acquired from 12 randomly selected spots on the Au–MXene substrates (10^−5^ mol/L). Table S1: Wavenumber and band assignments of the SERS spectra of the Au–MXene-PATP system based on 633 nm laser excitation; Table S2: Band assignment of carbendazim.
