# Supplementary material for: Photocatalytic Deposition of Au Nanoparticles on Ti3C2Tx MXene Substrates for Surface-Enhanced Raman Scattering
Source: Molecules. 2024 May 18;29(10):2383. doi: 10.3390/molecules29102383 (PMC11124034; doi:10.3390/molecules29102383)
Supplement: Supplementary file 1 [file molecules-29-02383-s001.zip › molecules-2978190-supplementary.pdf]

SUPPORTING INFORMATION

Photocatalytic deposition of Au nanoparticles on $Ti_3C_2T_x$ MXene substrates for surface-enhanced Raman scattering

Zhi Yang[1], Lu Yang[1], Yucun, Liu[1,]* and Lei Chen[1,2,]*

1 College of Chemistry, Jilin Normal University, Siping 136000, China; yangzhi12260102@163.com (Z.Y.); yanglu4818@163.com (L.Y.)

2 School of Materials Science and Engineering, Jilin Jianzhu University, Changchun 130118, China

* Correspondence: liuyc19873@163.com (Y.L.); chenlei82@jlju.edu.cn (L.C.)

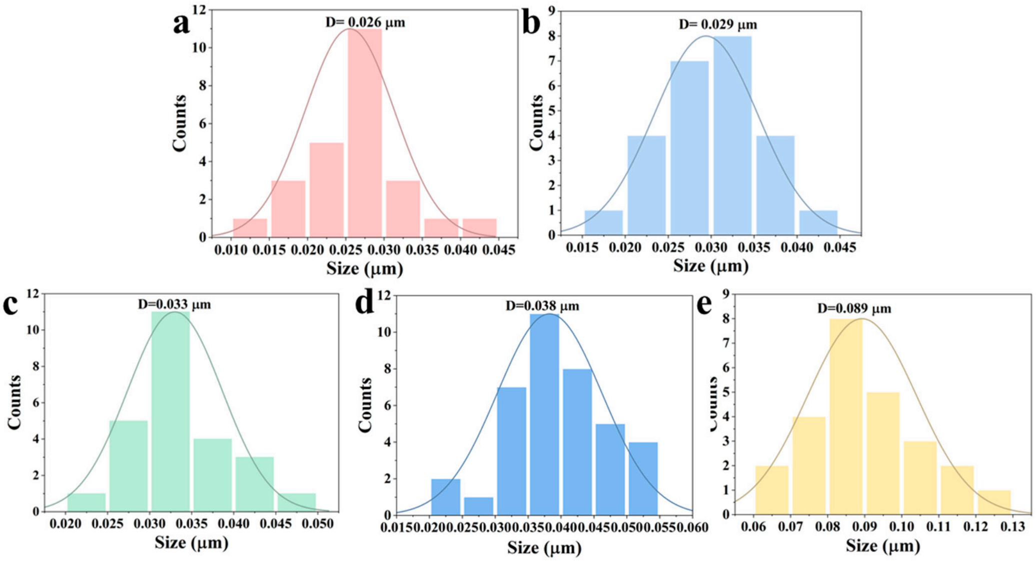

Figure S1. The size distribution of Au NPs absorbed on the surface of MXene nanosheets at different photoreaction time (10, 20, 30, 40, and 50 min).

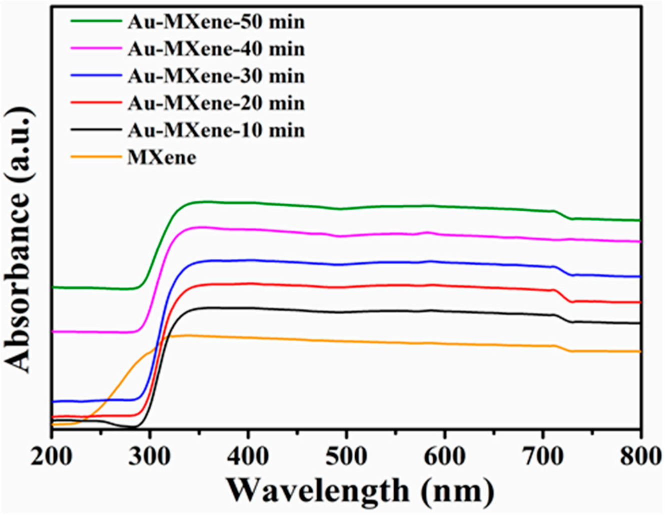

Figure S2. UV–Vis absorption spectra of MXene and Au-MXene composites (10, 20, 30, 40, and 50 min)

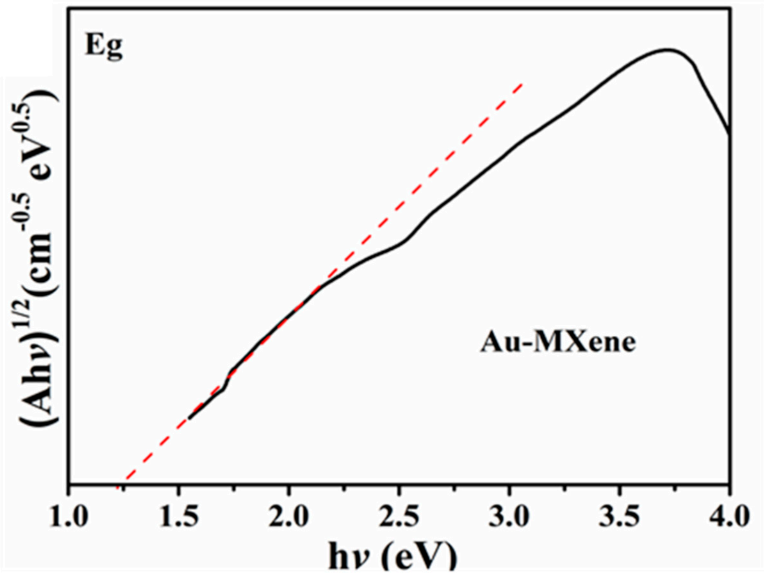

Figure S3. The plot of $(Ah\nu)^{1/2}$ versus photon energy (hν).

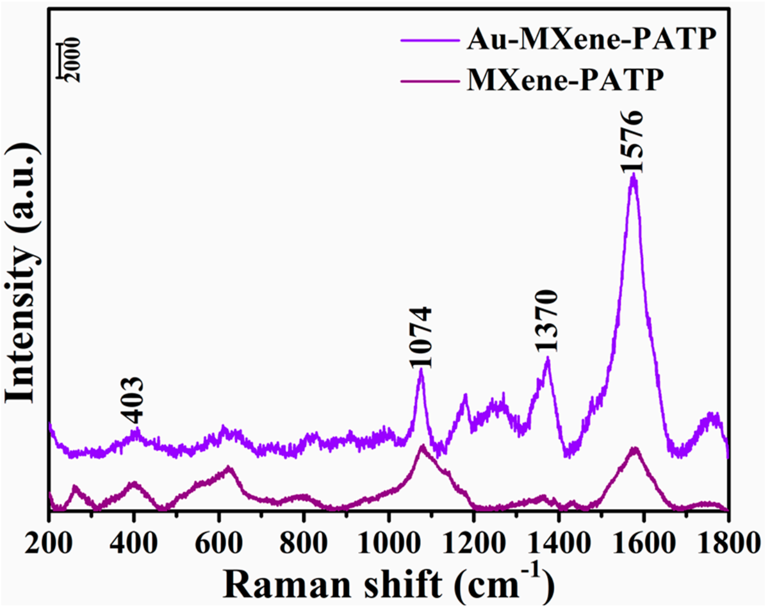

Figure S4. SERS spectra of PATP adsorbed on the MXene and Au-MXene composite.

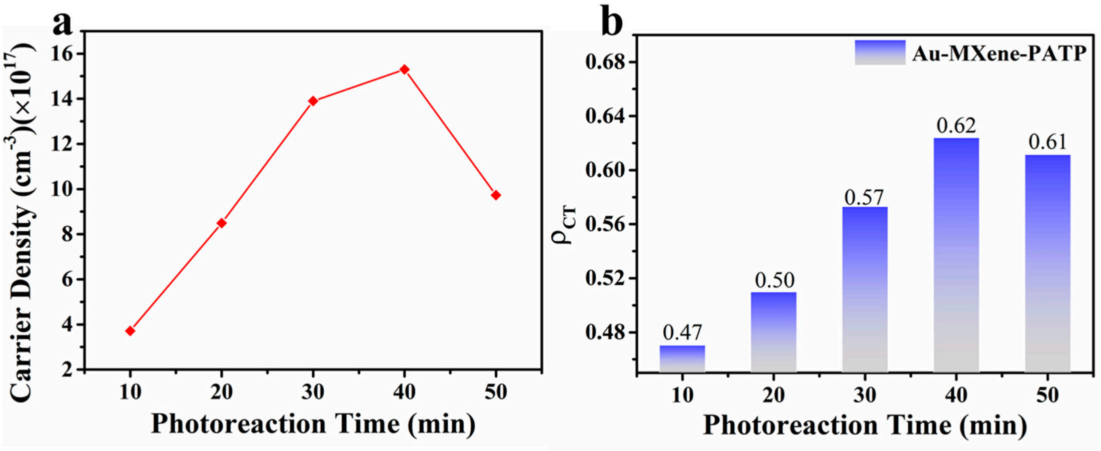

Figure S5. The photoreaction time of Au-MXene composites depends on the relationship between (a) carrier concentration and (b) charge transfer degree.

Table S1. Wavenumber and band assignments of the SERS spectra of the Au-MXene-PATP system based on 633 nm laser excitation[1].

**Wavenumber (cm^{-1})**	**Assignment**
403	O-H bending (MXene)
1074	C-S stretching (a1)
1180	C-H bending (b2)
1370	C-H bending + C-C stretching (b2)
1576	C-C stretching (a1)

The enhancement factor (EF) was calculated as follow:

$$EF = (I_{SERS}/N_{SERS})/ (I_{buik}/N_{bulk}) \quad (3)$$

Where I_{SERS} is the SERS signal intensity and I_{bulk} is the reference Raman signal intensity. N_{SERS} and N_{bulk} denote the number of molecules of the corresponding probe under laser irradiation, respectively.

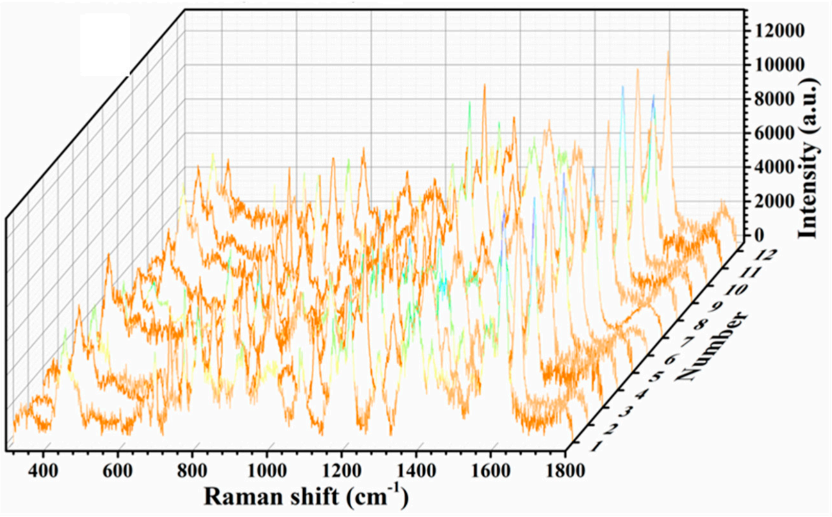

Figure S6. SERS spectra CBZ acquired from 12 randomly selected spots on the Au-MXene substrates (10^{-5} mol/L).

Table S2. Band assignment of carbendazim[2].

Solid Raman	SERS	Vibrational Description
655	680	Ring stretching and C–C bending
780	798	C–O–CH_3 bending
922	916	C–H bending
1021	1071	C–N bending
1228	1202	C–C bending and N–H bending
1274	1278	C–H stretching
1392	1380	C–N stretching
1618	1613	C=C stretching and C–C stretching

References

1. 1. Ji, R.; Sun, W.D.; Chu, Y. One-step hydrothermal synthesis of Ag/Cu_2O heterogeneous nanostructures over Cu foil and their SERS applications. *RSC Adv.* 2014; 4, 6055–6059.
2. Furini, L.N.; Sanchez-Cortes, S.; Lopez-Tocon, I.; Otero, J.C.; Aroca, R.F.; Constantino, C.J.L. Detection and quantitative analysis of carbendazim herbicide on Ag nanoparticles via surface-enhanced Raman scattering. *J. Raman Spectrosc.* 2015; 46, 1095–1101.
